# The Role of Rehabilitation after Spinal Mesotherapy in a Three-Stage Treatment Concept

**DOI:** 10.3390/jcm13113195

**Published:** 2024-05-29

**Authors:** Kamil Koszela, Michał Słupiński, Marta Woldańska-Okońska

**Affiliations:** 1 Department of Neuroorthopedics and Neurology Clinic and Polyclinic, National Institute of Geriatrics, Rheumatology and Rehabilitation, 02-637 Warsaw, Poland; 2 Rehabilika—Centre for Rehabilitation and Adult Age Medicine, 93-029 Lodz, Poland; 3 Department of Internal Medicine, Rehabilitation and Physical Medicine, Medical University of Lodz, 90-419 Lodz, Poland

**Keywords:** mesotherapy, local intradermal therapy (LIT), rehabilitation, conservative treatment, myofascial pain syndrome (MPS), risk factors

## Abstract

Sedentary lifestyles, work overload, and lack of regular physical activity are risk factors for spinal pain syndrome. In everyday medical practice, spinal pain syndrome of a muscular or myofascial, or non-neurogenic, nature is diagnosed. This problem affects a large group of patients and reaches about 70–80% of spinal pain cases. Usually, one of the primary treatments is with NSAIDs (Non-steroidal Anti-Inflammatory Drugs). In this case, one treatment method that is safe and has no side effects is spinal mesotherapy. This method consists of performing multi-point intradermal microinjections with the administration of drugs or medical devices. Based on a new perspective on the treatment of spinal pathology—the so-called three-stage treatment concept—it is necessary to deal with the risk factor(s) of spinal pain syndrome and reduce or at least modify them (stage I). This is followed by a broadly understood medical therapy, in this case spinal mesotherapy (stage II), which aims to relax tense tissues, improve mobility in the spine and thus reduce pain. As a result, conditions are created for the necessary process, which is rehabilitation in the broadest sense (stage III). Movement therapy, which is crucial in spinal pain syndrome, is performed with less pain, after obtaining better patient mobility. The purpose of this article is to evaluate the role of rehabilitation of patients after spinal mesotherapy in terms of the three-stage treatment concept for spinal pathology.

## 1. Introduction

Spinal pain syndrome can affect patients of different ages and can be caused by various pathologies. In most cases (70–80%) it is of an overload, muscular or myofascial nature. Such ailments are associated with a sedentary and over-stressful lifestyle and work, accompanied by a lack of regular physical activity. As known, the soft tissues surrounding the spine (muscles, tendons, ligaments and fascia) play an important role in stabilizing the spine. The era of electronics, smartphones, computers and other 21st century conveniences is not conducive to physical activity, and in fact limits it. The pursuit of career, money, and other socio-economic conditions force us to spend more hours at work. Activities are mainly performed in a sedentary position if it involves office work, or physical work where non-ergonomic use of the musculoskeletal system, including the spine, occurs, and the overloads generated are associated with the development of pain syndrome [1,2].

A new look at the problem of treating spinal pathology is the so-called three-stage treatment concept, which aims to take a comprehensive look at the global problem that is chronic spinal pain syndrome. This concept involves the following points [3]:

1. Assessment of risk factors, such as improper use of a cell phone (pressing the chin to the sternum generates a spinal overload of 25–27 kg, assuming a head weight of about 4–5 kg), setting the computer monitor at an incorrect height, sitting for hours at work (especially in a total kyphosis position), or lifting weights during physical work without prior motor preparation or performing a warm-up. Nowadays, reducing risk factors is very often impossible. Therefore, the doctor’s task is to assess the factors and at least modify them so that the loads on the musculoskeletal system, including the spine, are significantly reduced and properly directed [3,4,5].
2. Implementation of broadly understood medical therapy.
  Conservative as well as surgical treatment is applied, depending on the pathology. Analgesic, anti-inflammatory, myorelaxant pharmacotherapy is used. An increasingly common form of treatment is spinal mesotherapy, which aims to create the conditions for the third stage of the concept discussed. Very often in daily clinical practice, doctors encounter patients in the course of exacerbation of chronic spinal disease. As a result, there is increased tension of the soft tissues of the spine, restriction of mobility within the spine and, as a consequence, the development of pain of both muscular origin, musculo-fascial origin and facet syndrome (due to pathological displacement in the intervertebral joints, as a result of reflex muscle contraction, generating severe pain). The use of mesotherapy is intended to relax tense tissues, improve mobility and reduce pain, and ultimately improve daily function and quality of life [2,3].
3. After the patient has been adequately prepared during stage two, it is imperative to apply broad and comprehensive rehabilitation aimed at sustaining the effects of treatment.

The key is exercise therapy, which will improve circulation within the musculoskeletal system, and will stretch, relax, and strengthen the musculoskeletal apparatus. Also applicable is preparatory physical therapy as an element to improve the effectiveness of mesotherapy and the musculoskeletal organ for the planned exercise. During this stage, education of the patient, in terms of ergonomic use of the spine, is also an important aspect. It should also be emphasized that, after the application of stage two, ailments often subside (temporarily) and patients see no reason why they should continue with stage three. Therefore, it is also part of the doctor’s role to educate the patient [3,6].

The purpose of this article is to evaluate the role of rehabilitation of patients after spinal mesotherapy, especially in the light of the three-stage treatment concept for spinal pathology. Moreover, this article aims to motivate clinicians to conduct research in this area.

## 2. Risk Factors

The three-step approach to treating spinal pathology starts with assessing the risk factors causing the disease. We assess modifiable and non-modifiable factors. We make the patient aware that the non-modifiable factors are beyond our control, while those that can be modified should be modified, such as proper use of the cell phone, setting the computer monitor at the correct height, adjusting the workstation with spinal ergonomic principles and reducing body weight [3]. A study by Samartzis et al. published in 2011 [7] found that the presence of juvenile intervertebral disc degeneration was strongly associated with overweight and obesity, increased intensity of sacral pain and decreased physical and social functioning. Moreover, elevated BMI was significantly associated with increased severity of intervertebral disc degeneration. This study has public health implications for overweight and obesity and the development of lumbar intervertebral disc disease in young patients. Another study highlighted the importance of BMI in the context of spinal pain. The aim of an article, published in 2023 [8], was to examine the relationship between remote work and the incidence of back pain during the COVID-19 pandemic, and to analyze this relationship based on body mass index. In the study group, approximately one in four remote workers reported back pain during the COVID-19 pandemic. Taking into account the above factors, it is necessary to consider their modification in the planned treatment, especially if the therapy regimen is based on a three-stage treatment concept [3].

Nowadays, due to various situations such as socioeconomic issues, it may not be possible to eliminate the risk factor. Many times, during a medical consultation, the patient hears the doctor say ‘Please change your job’, because the factor causing the ailment should be changed. Can the patient adjust to such advice? If she is, for example, an accountant working many years in the profession, working many hours in front of a computer, sitting, it will be difficult for her to achieve earnings, job satisfaction and positive results by starting in a completely different position. In the opinion of the authors of this article, the key is to find such solutions so that the patient can continue in his profession, after first modifying the risk factors, implementing targeted medical therapy, in this case, for example, spinal mesotherapy, and then sustaining the effects, implementing targeted rehabilitation. Modification of factors in this case is key. Performing mesotherapy alone will not bring much effect if the factor causing the symptoms, which is the cause of the pathology, is still active [3].

## 3. Spinal Mesotherapy

Spinal mesotherapy is a minimally invasive medical procedure involving multi-point intradermal injection to a depth of about 3–4 mm.

Special thin needles with a diameter of 0.23–0.3 mm and a length of 4–13 mm (30–32 Gouce) are used during the procedure. The choice of needle will depend on the area of the procedure performed. In young patients and those with high levels of pain, thinner needles with a diameter of 0.23 mm will be used first; in older patients, standard needles with a diameter of 0.3 mm are preferred.

Normally, about 20–25 microinjections are performed within one spinal segment. The procedure in chronic pathologies is performed once a week and repeated a minimum of five times [9,10].

For each injection point, a deposit of the drug/medical device is administered at a volume of about 0.05–0.1 mL.

The mesotherapy regimen will depend on the affected area and the symptoms the patient will present. In the cervical segment of the spine, we can distinguish three schemes:

1. In local pain of the cervical segment: mesotherapy treatment is performed in the medial line and laterally about 1–1.5 cm from it.
2. In local pain with radiation of discomfort towards the occiput, the so-called cervicogenic syndrome, mesotherapy treatment is performed as in local pain, but injections are performed towards the occiput in the medial line and laterally about 1–1.5 cm from it on the right and left sides.
3. In local pain with radiation to the upper limb(s), a basic regimen is performed as for local pain plus mesotherapy to cover the supraspinatus region of the scapula.

There may also be such a clinical situation that, in addition to local pain of the cervical spine, the patient may have complaints radiating towards the occipital region, but also towards one or both upper limbs. Then we use the mesotherapy regimen as in points 2 and 3.

In the thoracic segment, mesotherapy is standardly performed laterally from the midline about 1–1.5 cm on the right and left sides.

In the lumbosacral region, similarly to the cervical, it is performed in the midline between the spinous processes, then laterally about 1.5–2 cm on the right and left sides, then about 5 cm laterally from the midline on the right and left sides [10].

It is also important to palpate the tissue before each injection, looking for the most painful points [10].

Applications include tropocollagen I, lignocaine, drug mixtures of NSAIDs with lignocaine, and others. The injections form microdeposits from which the drug (or drugs) is slowly released into the underlying tissues. Importantly, the drug is administered close to the site of pathology, allowing optimal therapeutic effect of drugs and puncture. Mesotherapy is characterized by a rapid onset of action, a prolonged local effect, and a drug-sparing effect. Topical intradermal therapy is based on the hypothesis that a drug injected into the superficial layer of the skin allows a longer pharmacological effect in the area of injection. This technique modifies the normal absorption kinetics of the injected drug; in particular, it slows systemic absorption and allows distribution in the tissues beneath the injection sites. Slow local distribution and longer drug persistence in the underlying tissues allow the use of lower drug doses and less frequent administration compared to the systemic route [9,11].

The primary goal of mesotherapy treatment is to relax tight soft tissues (muscles, fascia, ligaments, tendons), resulting in improved mobility and reduced pain.

Microinjection stimulates receptors in the skin and subcutaneous tissue, causing microinflammation and activation of repair mechanisms through an increase in inflammatory mediators. Stimulation of the circulatory, nervous and immune systems improves tissue blood flow. Myofascial pain syndrome (MPS) is associated with increased muscle and fascial tension, decreased physical activity, and consequent tissue fluid stasis and hyperemia [9,12].

Based on a review of the literature, mesotherapy is a safe form of treatment for musculoskeletal pathologies, including the spine, both acute and chronic in nature. As a rule, no undesirable changes are observed at the injection site. There may be transient redness and pain associated with the injection. During the mesotherapy procedure, the doctor deliberately palpates the affected soft tissue looking for the most painful points, the so-called trigger points, into which injections are also made. Depending on the desired drug, post-treatment pain may persist differently. When using lignocaine or a mixture of drugs with lignocaine, for example, the pain is generally short-term [13,14,15].

The mesotherapy technique is increasingly used by doctors. In addition to mesotherapy of the spine, this method is applied in the course of other pathologies of the musculoskeletal system, such as inflammatory lesions of muscle attachments to bone (enthesopathies, tendinopathies), where there is a standard increase in the tension of soft tissues, which causes pain and limitation of mobility [10].

## 4. Rehabilitation

The rehabilitation process is an integral step in the management of musculoskeletal pathologies, including those of the spine. It is very important to implement targeted rehabilitation in each specific pathology, in a timely manner [3,6].

The basis for ordering physiotherapy in myofascial conditions is the patient’s general condition, the doctor’s discernment of existing concomitant conditions and confirmation of non-neurogenic etiology. It is important to carry out an examination and function tests appropriate for a physiotherapy examination. The results obtained will provide information about musculoskeletal dysfunctions, balance and muscle strength disorders. Important for rehabilitation is preserved sensation, especially deep sensation. The feeling of pain informs about the severity of the disease and also predicts the time when symptoms will disappear. It is an important factor controlling therapy [16].

**Figure 1:**
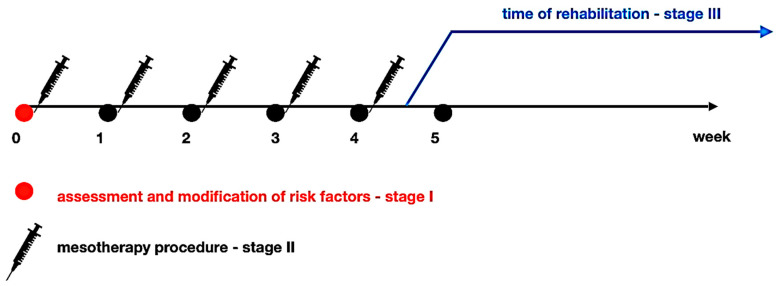
Mesotherapy and rehabilitation scheme in terms of the three-stage treatment concept.

Based on practical clinical observations, after about 4–5 mesotherapy treatments, physiotherapy management is recommended (Figure 1), which in the initial phase does not include exercises to strengthen the spinal muscles.

Physical therapy is particularly applicable as a factor that reduces pain, enhances the effect of pharmacotherapy and prepares for planned exercise. Because of the potential discomfort associated with it, kinesiotherapy is preceded, for example, by cryotherapy, which relieves pain and reduces inflammation, as well as facilitating a full range of motion. It is particularly noteworthy that minor side effects occurring after mesotherapy may be an exacerbation of an existing process, hence the anti-inflammatory and analgesic effects of physical therapy are particularly valuable for continuing the treatment process [17]. However, the timing of entry of physical therapy should be particularly carefully considered, as its effects may diminish the stimulus and pharmacological effects of mesotherapy.

An original type of therapy is the combination of TENS currents and mesotherapy described by Palermo et al. in cervicogenic pain syndrome [18]. A meta-analysis observed the results of low-energy laser therapy in patients with lower extremity tendinopathy, which were found to be promising in short-term follow-up [19]. It seems, however, that with the introduction of various forms of physical therapy, such as monochromatic light (LED) or laser radiation, one should wait until the interruption of mesotherapy treatments due to the possibility of photo-sensitization [20]. There are no contraindications to prior application of ultrasound or magnetic field. Moreover, a magnetic field facilitates absorption of the drug through the skin in magnetophoresis treatment [21].

Kinesiotherapy, introduced gradually, should be individual in nature. Depending on the patient’s condition and the severity of pain, one should start with breathing and isometric exercises and then move on to stretching exercises. Reducing muscle tension breaks the vicious cycle of pain and prevents aggravation of the disease process [3]. Once the contractures are removed and the muscle balance is restored, it is possible to proceed to strengthening the weakened muscle groups, while not exercising the antagonists that aggravate the pain.

Dry needling and manual therapy are particularly prominent in literature reviews for the treatment of myofascial pain, where the observed results are more positive than in placebo or comparison groups [22]. Another study applied transcutaneous electrical neuromuscular stimulation (TENS), hot compresses and ultrasounds to a control group. The study group had transverse massage applied with better results than the comparison group [17].

The study by Ay et al. shows that Kinesio Taping (KT) leads to a reduction in pain intensity, pressure pain threshold and range of motion of the cervical spine, but does not reduce disability in the short term. Therefore, Kinesio taping can be used as an alternative therapy for patients with myofascial pain [23]. However, Kinesio taping does not replace traditional physical therapy or exercise. On the contrary, KT may be most effective when used as an adjunctive therapy, perhaps by improving ROM, muscle endurance and motor control [24].

The physiotherapeutic methods cited above refer mainly to pain therapy for myofascial dysfunctions and have essentially no literature reference to physical therapy after mesotherapy. They are cited here by analogy as usually applicable after pharmacotherapy. Kinesiotherapy seems to be the obvious application, with much less potential for side effects or interactions. In addition to classical kinesiotherapy, methods such as yoga, Pilates or the McKenzie method combined with various types of breathing exercises may be applicable [16,25].

Therapy for spinal myofascial syndromes should be comprehensive and patient-directed. Cultural and social conditions should also be taken into account [16].

## 5. Discussion

Due to the fact that the literature on the role of rehabilitation after spinal mesotherapy is very limited, especially in terms of the three-stage concept of treating spinal pathology, with origins in early 2024, it is worth mentioning that this article presents a different perspective on the problem of spinal pathology of an overload, muscular or myofascial nature. Looking at statistics, the majority of patients with spinal pain complaints have pain of just such a nature. Therefore, it seems reasonable to use mesotherapy for this type of ailment, but medical therapy alone may not be enough. Therefore, in the three-step treatment concept, we first assess the risk factor(s) and modify them. The patient’s participation in the process is also important, as much depends on him. Then, after the implementation of medical therapy, in this case spinal mesotherapy, it is worth informing the patient that this mesotherapy is not the only solution but one of the stages of treatment, which takes several weeks. A patient who realizes this fact will better cooperate with the treating physician. In addition, patient education should be emphasized throughout the treatment process. Very often, the therapeutic effect after five mesotherapy treatments is very good and patients do not see the need to continue treatment. It is the doctor’s role to educate the patient and explain the important role of the rehabilitation process. Mesotherapy in this case, in addition to improving function and reducing pain, is a form of preparation for the rehabilitation process.

For more than 10 years, the literature has seen an increase in publications on the use, efficacy and safety of spinal mesotherapy treatments, but there is a lack of studies on the substitution of rehabilitation and the timing of its implementation after mesotherapy treatments. This article aims to make clinicians aware of the importance of improving a patient with spinal pathology after spinal mesotherapy and to mobilize them to conduct clinical research in this area.

Moreover, it is worth paying attention to the fact that mesotherapy is a relatively cheap and easy-to-perform treatment. Sometimes there may be a problem with the availability of mesotherapy, e.g., in Poland, mesotherapy is a partially known medical procedure, but it is not performed as widely as in Italy. The Italian Mesotherapy Society recommends mesotherapy for pathologies of the musculoskeletal system. There is no such scientific society in Poland, therefore international guidelines are necessary [10,12].

In recent months, several interesting papers on the efficacy and safety of spinal mesotherapy have appeared in the literature.

In a study by Scaturro net al. (2023) [26], as one of the few papers, the authors included a protocol for improving patients with exercise in addition to the use of mesotherapy of the cervical spine in fibromyalgia. On the basis of the study, they concluded that mesotherapy with diclofenac and thiocolchicoside is a safe and effective method of treating cervical spine pain in patients with fibromyalgia, in the short term, in terms of pain reduction, return to functional capacity and quality of life. In contrast, in this study, the timing of implementation of targeted physiotherapy was not evaluated.

Another study published in January 2024 by Ranieri et al. [27] reported on the use of mesotherapy for cervicobrachial pain syndrome with bilateral radiating to the arms/shoulders. This study found that mesotherapy with diclofenac sodium reduced pain intensity and improved functional outcomes, with no significant side effects in patients with myofascial pain syndrome located in the cervical/shoulder region. In contrast, this study did not consider the aspect of rehabilitation as a continuation of treatment according to the three-step treatment concept cited in this article.

A recently published paper by Koszela et al. [28] shows the efficacy and safety of collagen mesotherapy of the lumbar-sacral spine versus lignocaine 1%. In this study, the follow up period was 3 months. It turns out that, in addition to the injection therapy itself, the drug used plays an important role. In the longer follow-up, better results were obtained after tropocollagen I, despite the fact that the effects after lignocaine 1% mesotherapy were also satisfactory. This study did not consider the aspect of rehabilitation after injection therapy.

Therefore, looking at the overall problem of comprehensive treatment of spinal pain syndrome of overload, muscular or myofascial nature, it seems that rehabilitation plays a very important role in the three-stage treatment concept. It is highly important to plan a study to evaluate the condition of patients after the use of spinal mesotherapy in combination with rehabilitation, applied at different times of application. This article is also intended to encourage clinicians to carry out such studies by pointing out the gap in the literature in this area.

There are a growing number of reports in the literature showing the effectiveness of multimodal therapies for musculoskeletal pathologies, including the spine. In a study published in 2017 by Segura-Pérez M. et al. [29], the authors show the effectiveness of a multimodal rehabilitation protocol that included dry musculofascial trigger point needling, stretching, kinesio-taping, eccentric exercises and patient education. This study did not consider risk factors for myofascial pain syndrome.

Due to the global problem of spinal pain syndrome, many researchers are looking for solutions based not on a single therapeutic solution, but on more solutions, proposing different concepts. Steinmetz, publishing an article in 2022 [30], proposes new perspectives in the treatment of LBP. He stresses that more and more evidence shows that a more holistic and flexible approach is needed to individually diagnose and target the complexity of LBP.

Although many interventions can be used in combination, little is still known about the effectiveness of multimodal approaches. The purpose of the review by Liles et al. [31] was to examine outcomes comparing the combination of exercise and delivery of knee injections with exercise alone in the treatment of knee osteoarthritis. The literature obtained on multimodal therapy was insufficient. In the included studies, there was no better effect of combining knee joint injections with exercise, compared to exercise alone, for knee osteoarthritis, which is, however, of a different nature than myofascial pain syndrome.

In comparison, different results were obtained comparing groups of patients with osteoarthritis of the knees in the study by Chen et al. [32]. Better results were obtained in the group of patients in which physical therapy treatments, TENS currents, were added to hyaluronic acid injection. Improvements were observed in VAS pain intensity at 2 weeks of follow-up, as well as in the Lequesne index at 2 weeks and 3 months after therapy.

Although interdisciplinary multimodal pain management (IMPT) programs use a common biopsychosocial approach to improve the well-being of patients with chronic pain, significant differences in content and duration have been reported. Moreover, it is unclear to what extent beneficial health outcomes persist over time. The meta-analysis described by Elbers S. [33] shows that participation in an IMPT program is associated with significant improvements in well-being, which generally persist over the follow-up period without recurrence. The analysis included pharmacological, physiotherapeutic, psychotherapy and social interventions. The results indicate not only a reduction in pain and an improved physical and psychological state, but improved daily functioning and general well-being. The results also have a positive economic aspect.

Finally, it is worth mentioning patient education and self-management strategies as part of the rehabilitation process. An increasing number of analyses appear in the literature emphasizing the patient’s role in the recovery process [34,35].

In conclusion, the implementation of a functional musculoskeletal approach together with the emerging concept of nociceptive pain in individually targeted holistic approaches seems to be an effective way to deal with the complexity of LBP. Hence, it seems that the proposed three-step concept for the treatment of spinal pathology may be a good direction for physicians, physiotherapists and other specialists dealing with patients with spinal pain syndrome.

The limitations of this article include the lack of randomized controlled trials and meta-analyses on the role of rehabilitation after spinal mesotherapy. Nevertheless, this issue requires clinical studies on a resting group of patients in both a retrospective and prospective evaluation.

## 6. Conclusions

Based on the theoretical considerations and the authors’ experience, the rehabilitation process is an essential step in management after spinal mesotherapy. It should be included at the right time based on the three-stage treatment concept. Incorporating rehabilitation too early may result in a deterioration of treatment effects due to too rapid absorption of mesotherapy drugs/me products into the bloodstream and their lack of local effect.
